# Unraveling Sterilization Effects on Konjac Glucomannan: Insights into a Natural Biopolymer for Biomedical Applications

**DOI:** 10.1021/acsomega.5c11597

**Published:** 2026-01-20

**Authors:** Mariah Zajankauskas Orçati, Mariana Agostini de Moraes, Marisa Masumi Beppu

**Affiliations:** School of Chemical Engineering, Department of Materials and Bioprocess Engineering, 28132 University of Campinas, Campinas, SP 13083-852, Brazil

## Abstract

Sterilization is essential for ensuring the safety and biocompatibility of biomaterials intended for biomedical use. However, their sensitivity to sterilization methods requires careful evaluation of potential impacts on structural and functional integrity. Biopolymers such as konjac glucomannan (KGM) have emerged as promising candidates for wound healing and tissue engineering due to their favorable physicochemical properties along with improved thermal resistance and adequate mechanical properties. In this study, KGM-based films were developed and subjected to different sterilization and disinfection protocols—including autoclaving, ethylene oxide (EtO), γ irradiation, UV radiation, and 70% ethanol—and were evaluated on their physical, chemical, mechanical, and biological properties by scanning electron microscopy (SEM), Fourier-transform infrared spectroscopy (FTIR), X-ray diffraction (XRD), thermogravimetric analysis (TGA), mechanical analyses and in vitro cytotoxicity. The results showed that γ and UV irradiation induced the most pronounced changes in film properties, whereas autoclaving and EtO better preserved the materials’ integrity. Additionally, 70% ethanol demonstrated satisfactory performance as a disinfectant. These findings underscore the importance of selecting appropriate sterilization methods to ensure the efficacy and functionality of KGM-based biomaterials.

## Graphical abstract

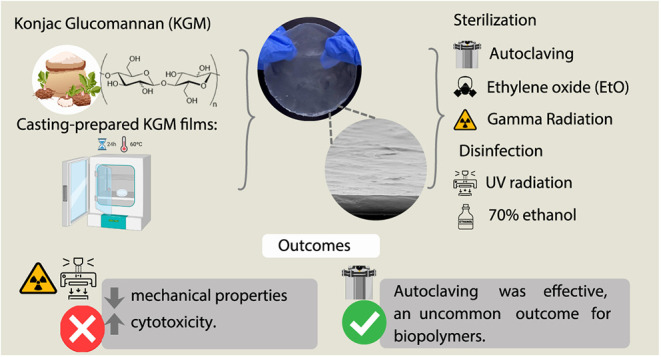

## Introduction

Research on biopolymers for the development of wound dressing matrices has intensified in recent years due to their favorable properties, such as biocompatibility, biodegradability, moisture retention at the wound site, and their ability to support tissue regeneration.^1^ Bioengineered biomaterials have shown significant potential owing to their capacity to mimic the natural extracellular matrix (ECM), providing structural support for cell proliferation, thereby accelerating wound healing, and reducing complications associated with skin regeneration.^2^

Among the biopolymers under investigation, konjac glucomannan (KGM) has attracted particular interest. KGM is a neutral polysaccharide extracted from the tuber of the *Amorphophallus konjac* plant, widely cultivated in East Asia and traditionally used in the food industry as a gelling, thickening, film-forming, and emulsifying agent.^3,4^ Glucomannan is composed of d-mannose and d-glucose units linked by β-(1→4) bonds in a 1.6:1 ratio, with approximately one acetylated glycosyl residue for every 19 units. The degree of acetylation is known to influence its solubility in aqueous solutions.^5,6^ Recent studies have highlighted the versatility of KGM for biomedical applications, including the development of films, membranes, hydrogels, and drug delivery systems.^7^

Owing to its hydroxyl- and acetyl-rich backbone, KGM is susceptible to a range of chemical and physicochemical modifications. These may include deacetylation under alkaline conditions, oxidative reactions leading to carbonyl or carboxyl group formation, and chain scission induced by physical treatments such as irradiation. Such processes can alter molecular weight, chain conformation and hydrogen-bonding networks.^8^ Its structure also promotes extensive interactions with water through hydrogen bonding and van der Waals forces, enhancing its absorption and retention capacity.^9,10^ Furthermore, modifications such as alkaline deacetylation can improve the thermal and mechanical properties of KGM, enabling the development of scaffolds with enhanced performance in aqueous environments.^7^

In this context, the present work focused on the development of KGM-based films using sodium hydroxide as a gelation agent, aiming to enhance their structural properties for biomedical applications.

However, to enable the clinical application of such biomaterials, ensuring sterility without compromising their physicochemical properties is essential. Sterilization methods must be carefully evaluated due to their impact on material integrity and product properties^11^ and must effectively eliminate all microorganisms, including bacterial spores.^12^ According to Kerwald et al.,^13^ sterilization remains a critical gap between laboratory findings and industrial feasibility. Common methods such as autoclaving, ethylene oxide treatment and γ radiation are widely employed, yet may induce undesirable structural changes, especially in biopolymers.^11,14,15^ γ and UV radiation are well-established methods for inducing cross-linking or degradation in some biopolymers,^11,16,17^ and thermal treatment, such as autoclave, can be problematic for biodegradable polymers with low glass transition temperatures,^18^ where steam exposure may lead to hydrolytic degradation.^19^

Sterilization of medical devices is governed by international standards, with validated protocols recognized by regulatory agencies such as the United States Food and Drug Administration (FDA) and the International Organization for Standardization (ISO). These regulations establish consolidated Class A sterilization methods for polymeric materials, including γ and electron-beam irradiation, ethylene oxide, dry heat, and steam sterilization, providing a robust basis for ensuring efficacy and safety.^20^ Therefore, assessing the effects of these methods on the material is essential before selecting an appropriate sterilization strategy.

In addition to sterilization techniques, disinfection methods such as 70% ethanol and ultraviolet (UV) radiation are also commonly employed, particularly during the laboratory development of such biomaterials. Ethanol promotes protein denaturation, cellular dehydration, and lipid dissolution, inactivating various microorganisms. However, it is ineffective against hydrophilic viruses and bacterial spores.^19^ Ultraviolet (UV) radiation is commonly used for disinfecting biodegradable scaffolds, inducing photoproducts that damage microbial DNA. While effective on vegetative bacteria, UV radiation has limited penetration and reduced efficacy against spores.^11,19,21^

Steam autoclaving, operating at 120–150 °C and 15 PSI, is effective and residue-free but may degrade polymeric biomaterials due to temperatures exceeding their Tg and melting points.^22,23^ Ethylene oxide (EtO) is widely used (around 50% of medical device sterilization) due to its compatibility with heat- and radiation-sensitive materials.^24^ However, EtO may alter structural and biochemical properties of biodegradable scaffolds,^19^ though its high penetrability makes it suitable for porous biomaterials.^14^

Ionizing radiation, particularly γ rays from Cobalt-60, sterilizes by ejecting valence electrons and generating reactive oxygen species (ROS), disrupting DNA and cellular components.^11,19,25^ Though effective against various microorganisms, including spores,^26^ γ irradiation may cause significant changes in chemical structure, mechanical properties, and degradation rates due to free radical formation.^19,27^

There is no universal sterilization method suitable for all biomaterials, as the effectiveness and impact of sterilization strongly depend on material composition and formulation. As discussed, biopolymers are generally susceptible to chain scission, and sterilization processes involving high temperatures or irradiation may induce cross-linking, molecular weight reduction, crystallinity loss, and the formation of oxidative byproducts. Therefore, sterilization strategies must be individually assessed to preserve the key physicochemical and functional properties of the material for its intended application.^11,13^ Current literature lacks studies investigating the effects of different sterilization methods on KGM materials. Existing works have focused on the use of γ radiation^28–32^ and autoclaving^33,34^ to obtain degraded glucomannan.

In this context, the present study aims to investigate the effects of different sterilization and disinfection methods (*autoclaving, ethylene oxide, γ radiation, UV radiation, and 70% ethanol*) on KGM-based films using sodium hydroxide as a gelation agent to identify safe and effective protocols that preserve the structural and functional features of the biomaterial, which are essential for its application as a potential wound dressing matrix.

## Materials and Methods

### Materials

KGM powder was supplied by *Herbal Island* (Utah) with an average molecular weight of 5.18 × 10^5^ Da and a polydispersity index of 1.02, which were measured by gel permeation chromatography. All procedures were carried out using ultrapure water, obtained from a Millipore filtration system, and all chemicals were used without further purification.

### Preparation of KGM Films

The film preparation protocol was adapted from the methodology proposed by Genevro et al.^35^ To obtain the films, the KGM powder was mechanically stirred with ultrapure water to prepare a 1% (w/v) solution. Then, sodium hydroxide (gelling agent) was added until a final concentration of 0.1 mol/L was reached. For film production, 40 g of the final solution was transferred to a polystyrene plate (*d* = 9 cm) and dried at 60 °C until reaching 1–2% of the initial mass. After this period, the samples were rinsed with ultrapure water until the washing solution reached a neutral pH. Finally, the films were stored in a refrigerator at 8 °C until analyses.

### Sterilization Methods

Samples were subjected to different sterilization (autoclaving, ethylene oxide and γ radiation) and disinfection (ethanol and UV radiation) methods to evaluate their effects in the physical, chemical, and biological characteristics of the material. The sterilization and disinfection methods and parameters were selected based on prior works that also evaluated the effects of different methods in biopolymer-based biomaterials.^23,36^

Autoclaving was performed at 121 °C for 15 min using a Phoenix Vertical Autoclave.

Ethylene oxide (EtO) sterilization was conducted at STERILENO (Araçoiaba da Serra, SP, Brazil) under controlled conditions of 145–150 kPa pressure, 5-h exposure time, and temperatures ranging from 50 to 55 °C.

γ radiation sterilization was carried out at Energy and Nuclear Research Institute (IPEN, São Paulo, Brazil) using a Cobalt-60 Multipurpose Irradiator at a constant dose rate of 4.33 kGy/h for 3 h and 30 min, totaling 25 kGy.

Ethanol disinfection involved immersing the samples in 70% ethanol for 48 h, followed by three washes with sterile phosphate-buffered saline (PBS) at pH 7.4.

For UV radiation, the films were exposed to UV light (λ = 254 nm) for 30 min on each side.

### Physicochemical Characterization

#### Scanning Electron Microscopy (SEM)

The surface microstructure was analyzed using scanning electron microscopy (SEM) with a Leo Electron Microscopy (Leo 440i – Cambridge, England). The samples were prefractured with liquid nitrogen, fixed onto the sample holder using double-sided carbon adhesive tape, sputter-coated with gold, and subsequently analyzed.

#### Fourier Transform Infrared Spectroscopy with Attenuated Total Reflectance (FTIR-ATR)

The investigation of the secondary structure and chemical composition of the films was carried out using a Fourier transform infrared (FTIR) spectrophotometer. The analysis was performed with an attenuated total reflection (ATR) accessory, covering the range of 675 to 4000 cm^–1^, with 4 cm^–1^ resolution and 128 scans.

### X-ray Diffraction (XRD)

To study the crystallinity of the sample, X-ray diffraction (XRD) analyses of the films were conducted using an X’PERT MPD, Philips Analytical X-ray (Almelo, Netherlands) instrument with Cu–Kα radiation (λ = 1.54 Å). The scanning rate was set to 0.6°/min, with a 2θ range from 5 to 80°. The crystallite size (*D*) and interplanar spacing (*d*) were calculated using the Scherrer ([Disp-formula eq1]) and Bragg ([Disp-formula eq2]) equations

1 Scherrerequation:D=Kλ/β cosθ

2 Bragg’slaw:d=λ/2sinθ

where λ is the X-ray wavelength, θ the diffraction angle, β the full width at half-maximum (FWHM) of the diffracted peak, and *K* the shape factor (set as 0.9 for polymeric materials). The relative crystallinity was determined from the crystalline area in the diffractograms using Origin software, according to eq 3

3 $$X_{c}=\left({F_{c}/(F}_{c}+F_{a}\right))\cdot 100\%$$

where *X*_c_ is the crystallinity, and *F*_c_ and *F*_a_ are the crystalline and amorphous areas, respectively.

### Thermogravimetry Analysis (TGA)

Thermogravimetric analysis (TGA) was used to evaluate the thermal behavior and stability of the films, as well as the degradation peaks of glucomannan. A TGA-50 M thermogravimetric analyzer (Shimadzu) was employed. The analysis was performed within a temperature range of 25 to 600 °C, with a heating rate of 10 °C/min and a nitrogen flow rate of 50 mL/min. The samples were dried prior to the analysis.

### Mechanical Tests

Tensile strength tests were adapted from ASTM D882-02 and performed using a TA.XT2 Texture Analyzer (Stable Microsystems SMD) with a 50 kg load cell. Films were cut into 7 cm (I) × 3 cm (*w*) strips and conditioned for 48 h at 50% relative humidity. Thickness (*e*) was measured with a digital micrometer (MDC-25S, Mitutoyo). To simulate a moist wound environment, samples were immersed in PBS (pH 7.4), and excess liquid was removed with filter paper prior to testing. After swelling, the films showed an approximate 200% increase in mass compared to their initial dry state.

Tensile testing was conducted with a 60 mm grip separation and a crosshead speed of 3 mm/s. Each sample was tested in five replicates, and the average values were calculated. Tensile strength (σ) and elongation at break (ε_b_) were determined using eqs 4 and 5, respectively, while Young’s modulus was calculated based on the linear portion of the stress–strain curve.

4 σ(MPa)=F/(e×w)

5 ϵb(%)=(d/l)×100%

where *F* is the applied force at break (N), *e* is the average thickness of the sample (mm), *w* is the sample width (mm), *d* is the displacement of the tensile tester grips at break (mm), and *l* is the initial gauge length (mm).

### In Vitro Cytotoxicity Test

The cytotoxicity test was performed to evaluate the harmful effects of materials or substances on living cells. In this study, the test followed the guidelines of ISO 10993-5 (2009) using the MTT assay on *L929* fibroblasts. The cells were cultivated in *Dulbecco’s Modified Eagle Medium* (DMEM) supplemented with 100 μg/mL penicillin and 100 μg/mL streptomycin at 37 °C under a 5% CO_2_ atmosphere for 24 h. KGM films (12 cm^2^) were immersed in 1 mL of supplemented medium and maintained at 37 °C for 24 h to obtain film extracts. Subsequently, 100 μL of 10^4^ *L929* cells and 100 μL of the film extracts were added to each well of a 96-well plate, which was incubated for 24 h before performing the MTT assay. This assay estimates cell proliferation by measuring the optical density (OD) of each well at 570 nm, as described in eq 6

6 Cell⁢viability(%)=(ODSample/ODControl)×100%

## Statistical Analysis

Statistical analyses were performed using *GraphPad Prism 8.0.2* to compare the samples nonsterilized, after sterilization and disinfection with autoclave, ethylene oxide, γ radiation, 70% ethanol, and UV radiation. Brown-Forsythe and *Bartlett’s tests* were used to assess the homogeneity of variances. *One-way ANOVA* followed by *Tukey’s multiple comparisons* test was applied to determine statistically significant differences among group means. Differences were considered significant at *P* < 0.05.

## Results

### Morphology before and after Sterilization

Figure 1 depicts the visual appearance of the films immediately after being processed through different sterilization protocols, in both dry and hydrated states. In the dry state, the films exhibit edge shrinkage. Notably, the autoclave treatment appears to enhance drying and promote the formation of roughness on the film’s surface. However, following the hydration process, the films regain their original conformation and handling resistance, with no visible differences between the samples. No differences in color or other physical characteristics were visually observed among the films.

**Figure 1:**
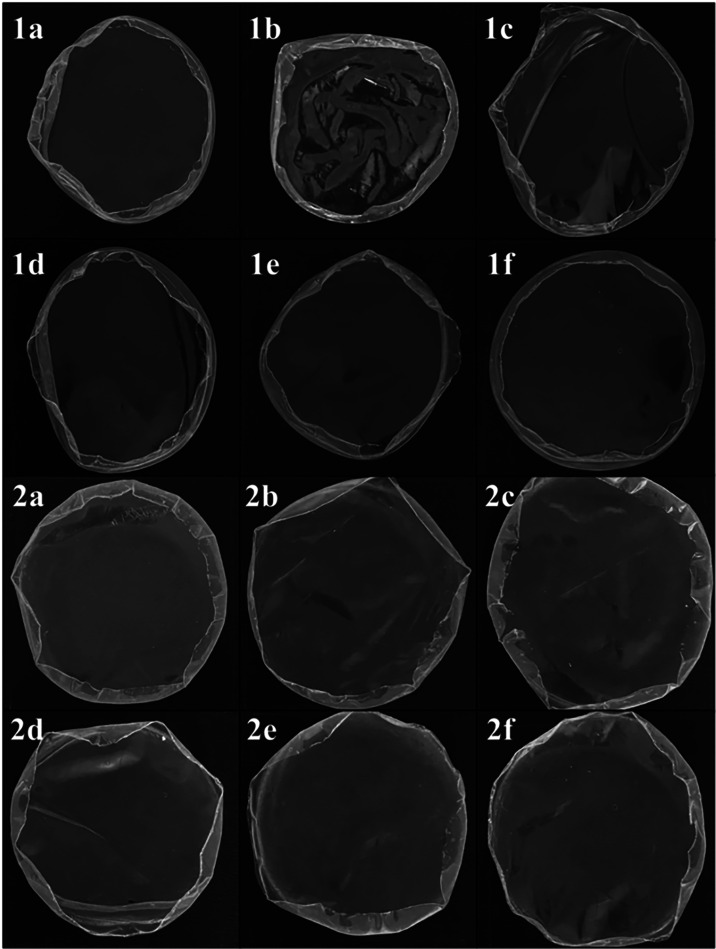
Photographs of dry (1) and hydrate (2) KGM films: nonsterilized (a), after sterilization with autoclave (b), ethylene oxide (c), γ radiation (d), 70% ethanol (e), and UV radiation (f).

SEM (Figure 2) was performed to analyze the morphology of KGM films and did not show morphological changes when comparing the different sterilization methods. In general, films exhibited a smooth, compact and homogeneous surface and cross-section, consistent with previous studies in literature.^37,38^ KGM possesses excellent film-forming properties and flexibility, contributing to these material characteristics.^39^ Surface roughness and localized air bubbles in the KGM films likely result from macromolecular entanglement and internal cohesion, leading to aggregate formation. The high viscosity of the solution also inhibited the release of air bubbles formed during heating, causing them to remain trapped within the matrix.^40^

**Figure 2:**
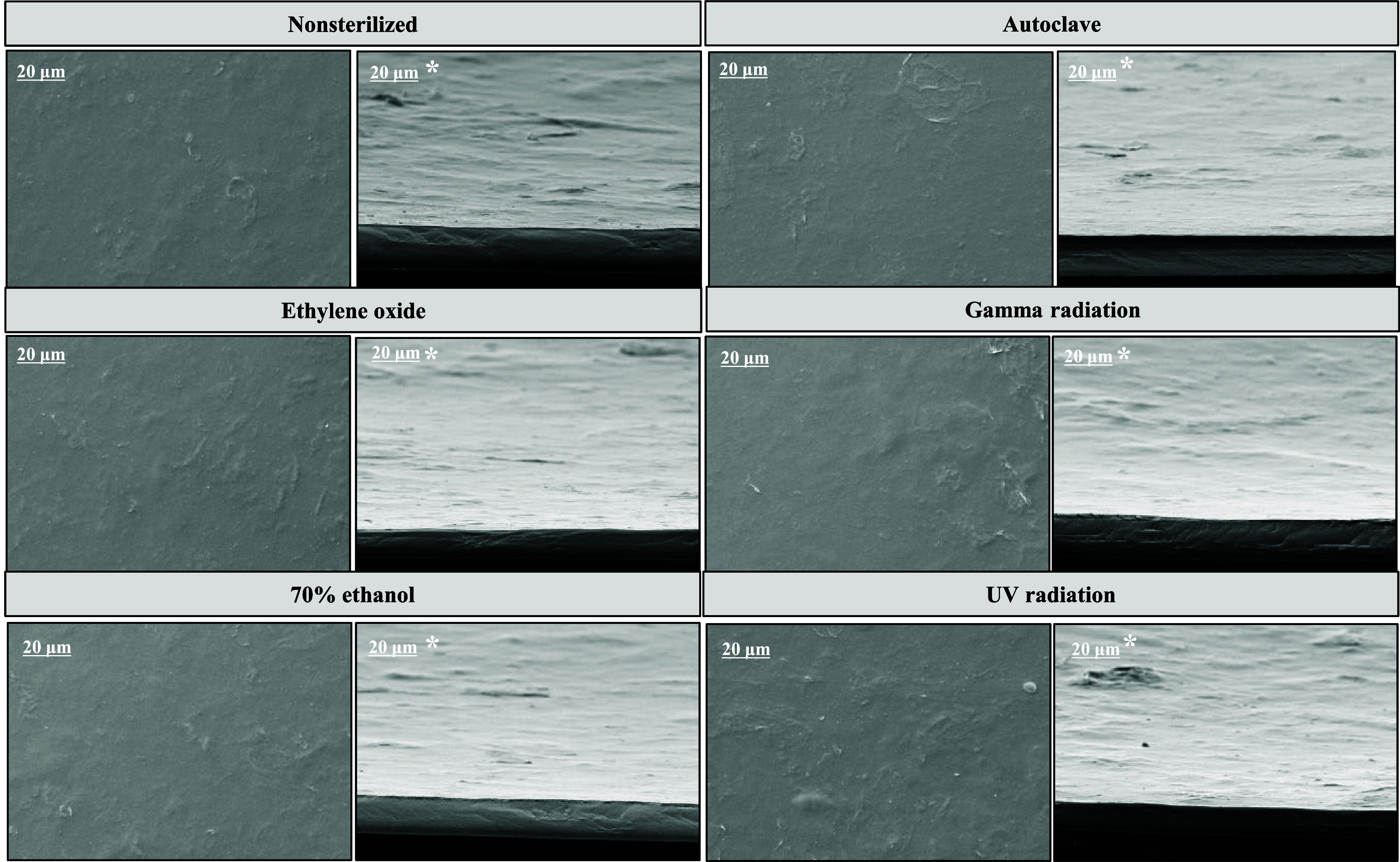
SEM images of KGM films submitted to different disinfection and sterilization methods. Images marked with * represent the cross section of the film.

### Chemical Structure

The spectra of the KGM films, before and after undergoing different sterilization and disinfection methods are shown in Figure 3. The analysis of the spectra revealed similar behavior among all samples, indicating that the different sterilization methods did not induce significant chemical alterations in the KGM films.

**Figure 3:**
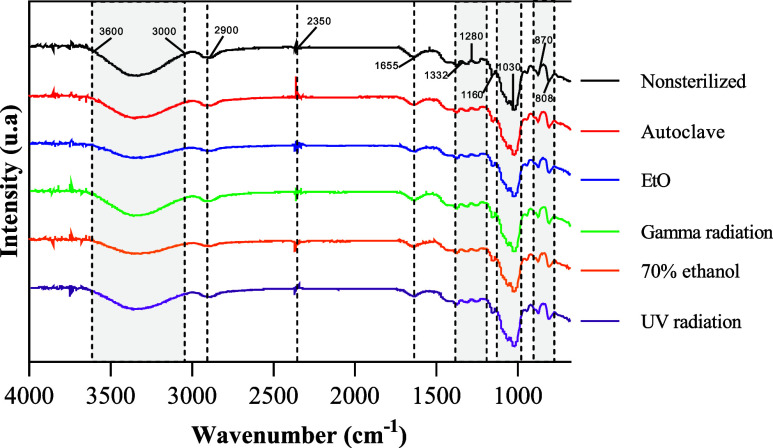
FTIR-ATR spectra of KGM films submitted to different disinfection and sterilization methods.

In all spectra, a broad band in the range of 3000 to 3600 cm^–1^ was observed, attributed to O–H stretching, along with a band around 2900 cm^–1^, corresponding to C–H stretching of the methyl groups present in KGM molecules. Additionally, the double absorption band around 2350 cm^–1^ is associated with atmospheric carbon dioxide (CO_2_).^41^ The band at 1655 cm^–1^ is associated with intermolecular hydrogen bonding, likely influenced by water absorbed in the KGM structure. Bands between 1280 and 1332 cm^–1^ were observed, corresponding to – OH bending and C = O stretching, while the bands near 1370 cm^–1^ are attributed to the angular deformation of the C–H bond. Peaks between 1030 and 1160 cm^–1^ indicate the presence of β-glycosidic linkages, and the characteristic absorption bands of mannose in KGM appear at 808 and 870 cm^–1^.^38,42–44^

A peak at 1730 cm^–1^, related to C = O stretching of acetyl groups in pure KGM^40^ was not observed in the film’s spectra due to NaOH treatment, indicating deacetylation—an essential process for the formation of the gel’s cross-linked structure.^45^

In conclusion, all films displayed similar FTIR profiles, with no new characteristic peaks, suggesting that the disinfection and sterilization methods did not induce evident chemical changes detectable by this technique. A slight reduction in the intensity of the broad O–H stretching band (3000–3600 cm^–1^), particularly in the ethanol and EtO treated samples, suggests partial dehydration of the polymer matrix or a reduction in intermolecular hydrogen bonding.^40^

### Crystallinity

About the film’s crystallinity, Figure 4 shows the diffraction patterns of the KGM films before and after sterilization.

**Figure 4:**
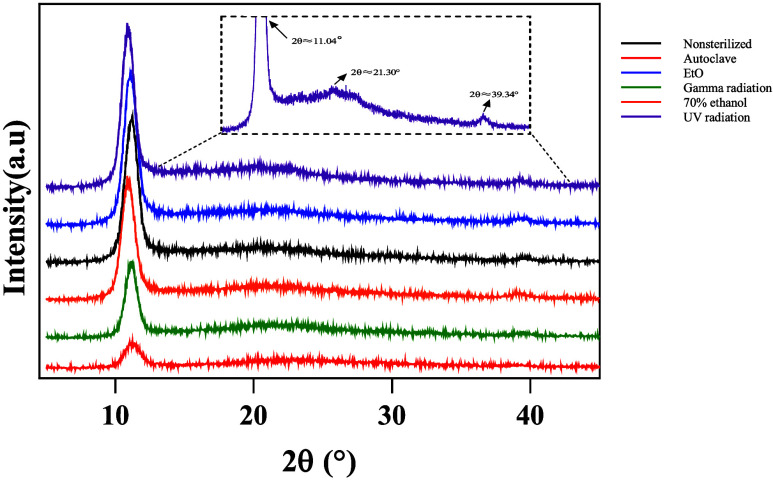
XRD diffractogram of KGM films submitted to different disinfection and sterilization methods. The inset highlights the broad halo around 2θ ≈ 21°.

All samples exhibited peaks and diffuse halos within the same angular regions, indicating a semicrystalline structure and aligning with previous studies that reported broad diffraction halos in the 11–22° range for KGM-based films.^42,46,47^

A more intense peak at 2θ ≈ 11.04° suggests regions of molecular organization, possibly related to the degree of glucomannan deacetylation.^46,48^ Deacetylation may enhance intermolecular hydrogen bonding, favoring the formation of ordered domains. Although structural refinement was not performed to define the crystalline system, previous studies report that deacetylated glucomannan can crystallize in the polymorphic mannan II form, with an orthorhombic unit cell, which is consistent with the present data.^49^

A diffuse halo at 2θ = 21.30° reflects the predominantly amorphous nature of the polysaccharide.^50^ Additionally, a weak peak at 2θ = 39.34° indicates intra- and intermolecular hydrogen interactions^51^ and the presence of tiny crystallinity.^40,42^

It is noticeable that a more intense diffraction peak at 2θ ≈ 11.04° is observed in this material, consistent with previous reports on KGM films subjected to alkaline treatment and different degrees of deacetylation. This feature suggests the presence of a predominant crystalline domain that is consistent with the structural organization reported for mannan II.^46,47^ Table 1 summarizes the structural parameters calculated from the dominant crystalline peak (2θ ≈ 11.4°), namely the interplanar spacing (d, Bragg’s law), crystallite size (D, Scherrer’s equation), and crystallinity degree.

**Table 1:** 2θ Values (°), Interplanar Spacing (Å), Average Crystallite Size (nm), and Crystallinity (%) of Each Sample

	2θ (°)	interplanar spacing (Å)	average crystallite size (nm)	crystallinity (%)
nonsterilized	11.23	7.86	6.95	44.05
autoclave	11.23	7.86	6.35	24.85
EtO	11.11	7.93	7.05	40.79
γ radiation	11.15	7.91	7.39	31.01
70% ethanol	10.85	8.14	7.03	41.50
UV radiation	10.85	8.14	7.45	35.26

The films exhibited typical semicrystalline behavior, with defined diffraction peaks and crystallinity values between 24.85 and 44.05%. The nonsterilized sample showed the highest crystallinity (44.05%), serving as the reference. γ- and UV-irradiated films displayed reduced crystallinity (31.01 and 35.26%), accompanied by slightly higher interplanar spacing (*d* = 7.91 and 8.14 Å) and crystallite size (*D* = 7.39 and 7.45 nm). These changes suggest that degradation predominated, leading to partial chain scission and loss of crystalline order. Although *d*-spacing remained nearly constant, the slight increase in crystallite size indicates that cross-linking in amorphous regions may have promoted local rearrangements, reflected in limited ordering of the residual crystalline domains.^17,52,53^

The ethanol-treated film exhibited a diffraction peak at 2θ ≈ 10.85°, a value closely matching the characteristic peak reported by Tong et al.^51^ for KGM gels equilibrated in ethanol (≈10.9°). In that study, ethanol exposure promoted partial dehydration of the KGM network and enhanced the aggregation of molecular chains, which in turn increased the degree of ordering and favored crystallization. A similar effect may explain the relatively higher crystallinity observed in our ethanol-treated films compared with the others treated samples, even though the crystallite size remained essentially unchanged.

In contrast, the autoclaved sample exhibited the lowest crystallinity (24.85%) and crystallite size (*D* = 6.35 nm). This pronounced reduction can be ascribed to the combined action of heat and moisture, which enhances chain mobility and disrupts pre-existing ordered regions, preventing the preservation or development of larger crystalline domains.^54^

### Thermal Properties

Thermal analysis results (Figure 5) indicated that KGM films exhibited comparable thermal behavior across all sterilization methods evaluated. The initial mass loss observed between 50 and 150 °C was attributed to moisture evaporation.^35^ A substantial mass loss, approximately 70%, was identified between 290 and 360 °C in the TGA curves, corresponding to the thermal degradation of the polymeric matrix, particularly the decomposition of saccharide rings and cleavage of glycosidic linkages.^55^

**Figure 5:**
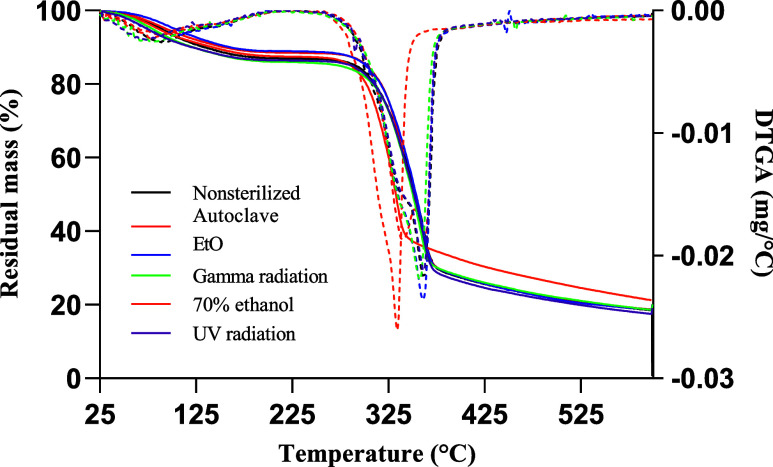
TGA and DTG thermogram of KGM films submitted to different disinfection and sterilization methods.

The differential thermogravimetric (DTG) analysis confirmed that the main degradation event occurred around 360 °C. However, samples treated with ethanol exhibited a slight shift in the onset of degradation, with decomposition initiating near 330 °C. The earlier thermal degradation in ethanol-treated KGM films may result from ethanol-induced dehydration, which reduces hydrogen bonding between polymer chains and weakens thermal stability. This is supported by the decreased intensity of the 3000–3600 cm^–1^ FTIR band, indicating lower water content. A similar behavior was also observed by Tong et al.,^51^ in KGM gels equilibrated in ethanol, where ethanol treatment led to reduced hydroxyl-associated bands.

Despite this marginal reduction in thermal stability, the values remain within the expected range for KGM-based materials. For instance, another work^56^ reported a decomposition temperature of 289.8 °C for ethanol purified KGM gels, further supporting the material’s favorable thermal resistance under the tested conditions.

### Mechanical Performance

The results summarized in the Figure 6 highlight statistically significant differences in the mechanical properties of KGM films subjected to various sterilization methods, including tensile strength, elongation at break and elastic modulus.

**Figure 6:**
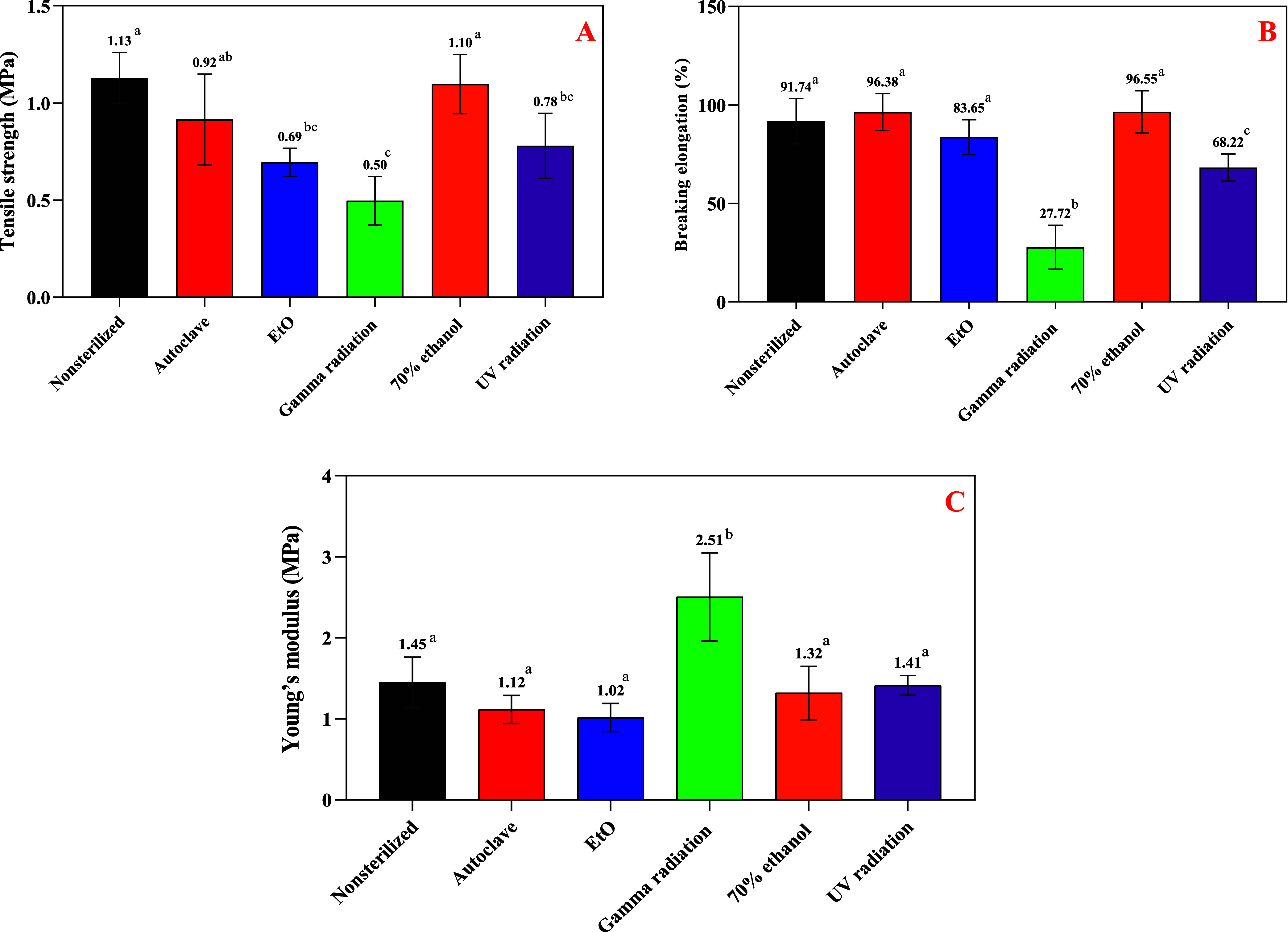
Mechanical test results of KGM films submitted to different disinfection and sterilization methods, where (A) Tensile strength, (B) Breaking elongation and (C) Young’s modulus. ^a,b,c^ Different letters in the same column indicate statistically significant differences (*p* < 0.05) between the means, according to Tukey’s test.

Regarding sterilization and disinfection methods, γ radiation had the most pronounced effect on the mechanical properties of KGM films, significantly decreasing tensile strength, elongation at break and increasing the elastic modulus, showing brittleness and stiffness material compared to nonsterilized. Previous studies by Xu et al., Prawitwong et al., and Jin et al.^29,30,32^ investigated the effects of γ irradiation on konjac glucomannan, demonstrating that irradiation induces dose-dependent chain scission with the formation of limited oxidative groups, while largely preserving the core polysaccharide structure. Consistently, all these studies reported a significant decrease in molecular weight upon γ irradiation, indicating that this approach is well established and widely suggested as an effective method for obtaining degraded KGM.

This literature also reports that such degradation may lead to the formation of small amounts of carbonyl groups and double bonds, contributing to structural loss and brittleness.^29^ However, the FTIR spectra obtained in this study did not reveal the appearance of new functional groups after sterilization treatments, including γ radiation. Xu^29^ reported, based on UV spectroscopy, the appearance of an absorption band at approximately 265 nm, which was attributed to the possible formation of carbonyl groups or unsaturated structures. According to that study, these structural modifications arise primarily from scission of the KGM main chain, involving the cleavage of glycosidic bonds during irradiation. Following chain scission, oxygen-centered radicals (O•) may either terminate by hydrogen abstraction or undergo molecular rearrangement, leading to the formation of carbonyl groups. Jin et al.^32^ similarly highlighted that γ radiation can directly break the main chains of polysaccharides such as KGM, producing smaller molecular fragments without necessarily generating new FTIR-detectable groups.

Moreover, even if the main chemical structure remains unchanged, irradiation can alter water interactions, affecting moisture retention and flexibility,^30^ consistent with the reduction in elongation at break observed for irradiated films. Altogether, these findings support the hypothesis that γ radiation induces structural degradation through chain scission.

The increase in Young’s modulus observed in γ-irradiated films may be related to the formation of stiffer cross-linked structures that restrict polymer chain mobility.^17^ This effect is consistent with XRD results, which suggest internal reorganization of the material. Thus, γ radiation appears to induce simultaneous degradation and cross-linking processes in the polymeric matrix.

Regarding UV radiation, it also reduced tensile strength and elongation at break, suggesting degradation as the underlying mechanism. Glucomannan consists of mannose and glucose units linked by β-1,4-glycosidic bonds and lacks strong chromophore groups, such as conjugated double bonds or aromatic rings. Consequently, its direct absorption of UV radiation is minimal, making it relatively resistant to direct photodegradation.^57^ However, in the presence of oxygen, UV exposure can initiate photo-oxidative processes, generating reactive oxygen species (ROS) capable of attacking polymer chains,^57^ and this indirect mechanism may lead to chain scission and changes in physical properties.

Notably, the autoclaved films exhibited good mechanical performance. In the literature, Tg values of KGM vary depending on formulation, with pure glucomannan films showing Tg around 113 °C.^58^ For the autoclaved samples, drying prior to sterilization may have contributed to an increase in Tg, as water acts as a plasticizer in polysaccharides, reducing Tg by enhancing chain mobility.^59^ The satisfactory mechanical behavior, despite reduced crystallinity, suggests that the material’s Tg is close to or above the sterilization temperature (121 °C). This indicates that the films retained a dense polymer structure with restricted chain mobility—likely influenced by deacetylation and prior drying—providing greater thermal and mechanical stability during moist heat sterilization and mitigating hydrolysis even above the estimated Tg.

### In Vitro Cytotoxicity Test

Figure 7 presents the results of the indirect cytotoxicity tests of KGM films before and after being subjected to different disinfection and sterilization methods. For this assay, culture medium extracts of the KGM films were prepared and brought into direct contact with fibroblast cells for 24 h.

**Figure 7:**
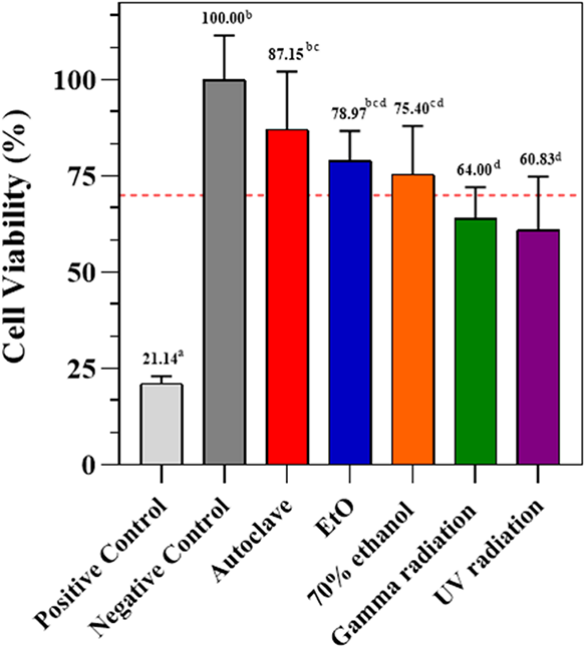
Cell viability of KGM films submitted to different disinfection and sterilization methods. The red line indicates 70% cell viability, in accordance with ISO 10993-5. ^a,b,c^ Different letters in the same column indicate statistically significant differences (*p* < 0.05) between the means, according to Tukey’s test.

According to ISO 10993-5, if cell viability at the highest concentration of the sample extract exceeds 70%, the material can be considered noncytotoxic. It was observed that the films sterilized by γ and disinfected by UV radiation had values below the threshold established by the standard. Both UV and γ radiation can induce structural modifications in polysaccharides, and these changes may also increase cytotoxicity due to the formation of reactive compounds and unfavorable cell interactions.^17^ Supported by the discussed mechanisms of degradation associated with UV and γ radiation, it is possible that reactive oxygen species (ROS) were released into the extract medium, contributing to the observed cytotoxicity.^17^

Pan et al.,^31^ demonstrated that the degradation of konjac glucomannan (KGM) by γ-ray irradiation, particularly under oxidizing conditions, is an effective strategy for producing KGM oligosaccharides. The degradation mechanism has been attributed to the direct interaction of high-energy photons with the polymer backbone, leading to radical formation and chain scission (R + hν → R•; R• → R_1_ + R_2_), in agreement with previous reports.^29,30^

These radical species may induce the cleavage of β-(1→4)-glycosidic linkages via molecular rearrangements, leading to the formation of carbonyl-containing fragments. Such carbonyl groups may represent potential sources of reactive carbonyl species, which are highly reactive toward cellular components and have been associated with cytotoxic effects and cellular damage in biological systems.^60^ Consistently, studies on oxidized polysaccharides, such as alginate and hyaluronic acid, have demonstrated that higher degrees of oxidation, combined with increased aldehyde content and reduced molecular weight, result in a marked decrease in cellular metabolic activity and pronounced cytotoxic effects in fibroblasts, which have been attributed to carbonyl-containing degradation products.^61^

## Conclusion

KGM films demonstrated significant structural, chemical, and thermal stability following exposure to various sterilization and disinfection methods. Among the treatments evaluated, γ and UV radiation resulted in the most pronounced degradation, with impacts on mechanical properties, cell viability and crystallinity. Results for γ-irradiated films indicate simultaneous degradation and partial cross-linking. Irradiation primarily induced chain scission, reducing crystallinity, while limited cross-linking in amorphous domains contributed to increased stiffness and restricted reorganization of crystalline regions. Future studies including molar mass analysis by gel permeation chromatography (GPC) could help strengthen this hypothesis.

In contrast, autoclaving and ethylene oxide (EtO) sterilization were the most effective in preserving the integrity of the material. Although EtO is widely used due to its compatibility with a range of materials, its application must be carefully considered given its high toxicity and environmental risks. Autoclaving, by contrast, is a well-known, safe, and cost-effective method that maintained the structural and functional properties of KGM films, even under conditions that typically degrade polysaccharide-based materials. This performance may be related to a comparatively higher glass transition temperature (Tg), which could enhance their thermal and mechanical stability during moist heat sterilization.

Additionally, despite being a disinfection rather than sterilization method, ethanol treatment preserved the films’ characteristics to a considerable extent. This suggests its potential applicability in contexts where disinfection is sufficient.

Overall, the findings support the use of autoclaving as a preferred method for sterilizing the KGM films developed in this study. As a next step, it would be important to evaluate the effectiveness of autoclave sterilization on this material by directly assessing microbial inactivation.

## Data Availability

All data can be requested from the corresponding author. M.Z.O.: investigation (lead), data curation (lead), formal analysis (lead), writing – original draft (lead). M.A.d.M.: supervision (supporting), conceptualization (supporting), writing – review and editing (equal). M.M.B.: conceptualization (lead), funding acquisition (lead), supervision (lead), writing – review and editing (equal).
